# Real-Time Self-Regulation of Emotion Networks in Patients with Depression

**DOI:** 10.1371/journal.pone.0038115

**Published:** 2012-06-04

**Authors:** David E. J. Linden, Isabelle Habes, Stephen J. Johnston, Stefanie Linden, Ranjit Tatineni, Leena Subramanian, Bettina Sorger, David Healy, Rainer Goebel

**Affiliations:** 1 Institute of Psychological Medicine and Clinical Neurosciences, Cardiff University School of Medicine, Cardiff, United Kingdom; 2 School of Psychology, Bangor University, Bangor, United Kingdom; 3 Department of Cognitive Neuroscience, Maastricht University, Maastricht, The Netherlands; 4 School of Social Sciences, Brunel University, Uxbridge, United Kingdom; 5 Institute of Psychiatry, Kings College, London, United Kingdom; 6 Mental Health Services, Betsi Cadwaladr University Health Board, Bangor, United Kingdom; Bellvitge Biomedical Research Institute-IDIBELL, Spain

## Abstract

Many patients show no or incomplete responses to current pharmacological or psychological therapies for depression. Here we explored the feasibility of a new brain self-regulation technique that integrates psychological and neurobiological approaches through neurofeedback with functional magnetic resonance imaging (fMRI). In a proof-of-concept study, eight patients with depression learned to upregulate brain areas involved in the generation of positive emotions (such as the ventrolateral prefrontal cortex (VLPFC) and insula) during four neurofeedback sessions. Their clinical symptoms, as assessed with the 17-item Hamilton Rating Scale for Depression (HDRS), improved significantly. A control group that underwent a training procedure with the same cognitive strategies but without neurofeedback did not improve clinically. Randomised blinded clinical trials are now needed to exclude possible placebo effects and to determine whether fMRI-based neurofeedback might become a useful adjunct to current therapies for depression.

## Introduction

Depression is the mental disorder with the largest impact on public health. Up to 20% of the population suffers from a depressive episode at some point in their lives [1], and major depressive disorder (MDD) is a main source of disability for adults of working age in industrialized countries. At least 30% of patients with MDD do not respond to standard pharmacological and/or psychological treatments [2], and a considerable number of those who do respond initially go on to develop a chronic relapsing-remitting disorder. These patients with no or only a partial response to standard treatments often enter a vicious circle of psychosocial decline with further deterioration of their mood and level of functioning. To prevent relapses new therapeutic strategies have to be developed that aid the restructuring of cognitive schemas and might even prevent the formation and crystallization of dysfunctional thought patterns during early phases of depression.

Over the last two decades, several new treatment techniques have been developed that were at least partly motivated by neuroimaging findings. These invasive [3] and non-invasive brain stimulation techniques [4] target the neural circuits believed to be involved in the maintenance of dysfunctional cognitive patterns and to change their activity in response to treatment [5], [6]. Although two new stimulation techniques (Vagus Nerve Stimulation, VNS; Transcranial Magnetic Stimulation, TMS) have received FDA approval, one of them requires a surgical procedure (VNS) and the other (TMS) has had mixed clinical effects [4]. Moreover, even the most effective stimulation technique–electroconvulsive therapy (ECT)–has only relatively short-lived effects [7]. The alternative, or complementary approach of teaching patients strategies that would eventually become self-sustainable has traditionally been the domain of cognitive behavioral therapy (CBT). Although CBT has recently been linked with neuroimaging to assess its neural effects [5], [6], neuroimaging findings have not directly been integrated in the therapeutic process.

Here we report a proof of concept study for a neuroimaging-based technique that tries to achieve such integration by combining concepts from brain stimulation, cognitive restructuring and emotion regulation research. This technique, functional magnetic resonance imaging (fMRI)-based neurofeedback, entails training patients to regulate their emotion circuits themselves through neurofeedback. The continuously updated neurofeedback signal shows the activity level in the targeted area, thereby providing patients with online information about their success in regulating their own brain activity.

Several studies have demonstrated that healthy participants [8]–[11] and patients with schizophrenia [12] can learn self-regulation of brain areas involved in emotion processing through real-time feedback of local fMRI signals, and successful self-regulation was associated with altered appraisal of aversive stimuli [13]. The first clinical application of fMRI-based neurofeedback in patients with chronic pain has been promising. In this study, successful self-regulation of activity in the anterior cingulate cortex, an area involved in the affective processing of pain, was associated with a reduction in pain ratings [14]. In the present study we localized areas responsive to positively valenced visual stimuli adapted from the International Affective Pictures System (IAPS) [15], [10] and then trained patients with unipolar depression to upregulate the activity in this target region over four sessions. We hypothesized that the combination of the physiological upregulation and the reinforced training of positive thought patterns would lead to an improvement of mood, which would not be seen in a control group that engaged in an emotion regulation protocol without neurofeedback.

## Materials and Methods

### Participants

Eight patients with a DSM-IV diagnosis of major depression (Recurrent Depressive Disorder: 296.3) and no co-morbid DSM-IV pathology were recruited from outpatient clinics into the experimental (neurofeedback: NF) group. We subsequently recruited a control group to undergo an imagery (IM) procedure outside the scanner to control for non-specific effects of study participation and emotional imagery. Both groups were approached by their clinicians about their interest in participating in a research study exploring the effects of new treatments for depression. A psychiatrist (S.L.) confirmed each patient’s diagnosis using a clinical interview based on the Structured Interview for DSM-IV (SCID). All participants were recruited from the same clinics and had to adhere to identical criteria, and the two groups did not differ in mean age (NF = 48.38 years; IM: 48.5 years; *t*(14) = −0.18, *p* = .99), duration of illness (NF = 19.25 years, IM = 19.15 years; *t*(14) = 0.19, *p* = .99), or handedness (one left-handed individual in each group), but there were three females in the IM group and only males in the NF group. All patients had been on a stable dose of antidepressant medication for at least six weeks preceding the intervention. The groups were comparable in terms of their drug treatment. Six NF patients were treated with antidepressants (AD) only; one, with AD and lithium; and one, with an AD and an antipsychotic. Seven IM patients were treated with an AD only; and one with an AD and lithium. For additional details, see Table 1.

**Table 1:** Patient demographic characteristics.

No.	Age	Gender	Handedness	Duration of illness (years)	Medication (daily doses)
NF group					
1	54	M	L	1	lofepramine 140 mg, mirtazapine 30 mg
2	67	M	R	49	amitriptyline 75 mg
3	37	M	R	6	tranylcypromin 40 mg, lithium 400 mg
4	21	M	R	2	fluoxetine 40 mg
5	44	M	R	20	mirtazapine 30 mg
6	56	M	R	20	sertraline 200 mg, reboxetine 8 mg
7	47	M	R	25	citalopram 60 mg, quetiapine 100 mg
8	61	M	R	31	fluoxetine 20 mg
IM group					
9	39	M	R	20	duloxetine 60 mg
10	59	M	R	9	lithium 1200 mg, venlafaxine 225 mg
11	65	F	R	40	sertraline 100 mg
12	49	M	R	20	reboxetine 4 mg
13	64	F	R	12	citalopram 20 mg
14	29	M	L	18	citalopram 30 mg
15	44	F	R	20	citalopram 20 mg
16	39	M	R	14	citalopram 40 mg

### Ethics Statement

The research was conducted in accordance with the Declaration of Helsinki and all patients gave informed written consent before taking part in the study. The study received approval from the ethics committees of the School of Psychology, Bangor University, and the North West Wales NHS Trust. Patients received a monetary compensation of £10 per hour for their time and effort. All patients were debriefed about their individual strategies and about potential distress upon completion of the post-intervention assessments. Because it employed an experimental rather than clinical trial design the study was not registered in a public trials database.

### General Procedure

All patients completed an initial testing session that included the clinical interview, an assessment of their depression with the HDRS, their reward sensitivity (Behavioural Inhibition System and Behavioural Activation System (BIS/BAS) [16]), and their metacognitive dispositions (Thought Control Questionnaire (TCQ) [17] and Thought Control Ability Questionnaire (TCAQ) [18]). The first experimental session immediately followed. Each session started and ended with an assessment of the patient’s current mood using the Profile of Mood States (POMS [19]) and the Positive Affect Negative Affect Schedule (PANAS [20]). Immediately following the pre-intervention assessment, the patients in the NF group underwent the fMRI-neurofeedback procedure and the IM group performed the matched imagery. The second, third, and fourth experimental sessions were given at 1–2 weekly intervals during a period of 4–6 weeks. Immediately after the fourth session, the HDRS was administered again.

### Description of Psychometric Tests

#### Measures of depression and current mood state

The main outcome measure of clinical effects was the 17-item HDRS, which is a clinical rating scale that captures core components of the depressive syndrome and a standard measure of treatment outcome. We administered the full 21-item HDRS through a standardized interview by a board-certified psychiatrist (one of co-authors D.L., D.H., R.T., or S.L.). The sequential group allocation we had to implement made it impossible to blind the psychiatrists. We also assessed effects on current mood state with the POMS and PANAS before and after each session. On the POMS Standard Form [20], patients circle the number that correspond to their current mood state (0 =  “not at all”, 1 =  “a little”, 2 =  “moderately”, 3 =  “quite a bit”, 4 =  “extremely”) on sixty-five items. The higher the score that is obtained on this test, the greater the Total Mood Disturbance (TMD) that is experienced, and a drop in TMD was expected after each neurofeedback session. The POMS has satisfactory test-retest reliability, and internal consistency is satisfactory [21]. The PANAS is a self-report scale that consists of 20 items that state 10 positive and 10 negative feelings or emotions, which have to be scored on a scale from 1 to 5 (1 =  “very slightly or not at all”, 2 =  “a little”, 3 =  “moderately”, 4 =  “quite a bit”, 5 =  “extremely”). It was expected that the scores on the positive scale would increase after a neurofeedback session and those on the negative scale would decrease. It has high internal consistency and high factorial, convergent, and discriminant validity [19].

#### Measures of reward sensitivity and thought control

The BIS/BAS questionnaire [16] consists of 24 statements that belong to one of the four scales: BIS, BAS reward responsiveness, BAS drive or BAS fun seeking. The participants scored these statements on a scale from 1 to 4 (1 =  “very false for me”, 2 =  “somewhat false for me”, 3 =  “somewhat true for me, 4 =  “very true for me”). This questionnaire was administered at the beginning of the experiment to obtain an indication of control over motivational behaviour. A high score on the BIS scale is associated with sensitivity to signals of punishment which causes the inhibition of goal achievement. High scores on the BAS scales on the other hand imply sensitivity to signals of reward, which leads to a reinforcement of goal-directed behaviour. The BIS/BAS scales have good internal reliability and factor validity [22]. The TCQ consists of 30 sentences which describe a certain strategy that can be adopted when one experiences an unpleasant or unwanted thought and measures five factors: reappraisal, distraction, punishment, social control and worry. For each strategy patients marked whether they “never”, “sometimes”, “often” or “almost always” engaged in that particular strategy upon experiencing intrusive thoughts. A high score on the TCQ implies that the respondent adopts more adaptive strategies to control his or her thoughts. It has an acceptable test-retest reliability [17]. The TCAQ is composed of 25 statements that give an indication of how well patients are in suppressing unwanted thoughts [18]. Patients rated these statements on a 5-point Likert scale (“strongly disagree”, “disagree”, “neutral or don’t know”, “agree” or “strongly agree”). A higher score on the TCAQ is associated with a greater perceived control over one’s intrusive thoughts. The TCAQ has high internal consistency and retest reliability [23]. We obtained these measures to ensure that any group differences in clinical outcome were not produced by baseline differences in reward sensitivity or perceived self-control.

### FMRI Procedure (NF group)

**Figure 1:**
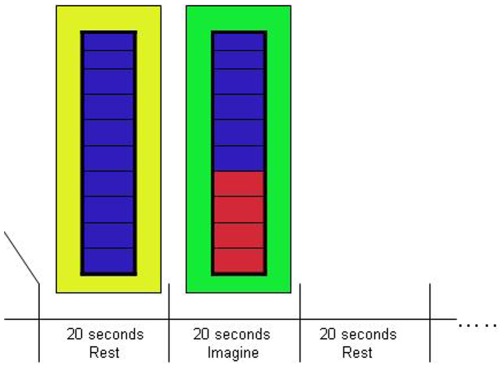
Neurofeedback protocol. During the neurofeedback runs (3 in each of the 4 sessions), participants alternated between 20 s periods of rest and 20 s periods where they had to upregulate activity in the target area. The level of activation was fed back in real time (updated for each TR of 2 s) through the thermometer display.

Patients in the NF group were trained to upregulate brain areas responsive to positive emotions using a procedure modeled on our previous work with healthy participants [10]. A target area was identified by the contrast between responses to positive and neutral images in a localizer scan to ensure that an area involved in positive emotion processing was selected. In the localizer scan, we assessed brain responses to positive, negative and neutral pictures by presenting four pictures of the same emotion category in blocks of 6 s (1.5 s per picture), alternating with a fixation baseline of 12 s. We presented 12 blocks per category in pseudorandom order. We used pictures from the IAPS [15] with negative (mean normative ratings for valence 2.8 [SD.42], arousal 5.63 [SD.55]), positive (valence 6.90 [.55], arousal 6.00 [.74]) and neutral valence (valence 5.45 [.56], arousal 3.44 [.47]). Pictures showed, for example, scenes of danger or disgust in the negative category, and scenes of romance including mild erotica or exciting sports in the positive category. After the localizer scan, patients were trained to upregulate the target area during three neurofeedback scans lasting ca. 7 minutes each per session (Fig. 1). Patients were informed about the general function of the target area but were not given any specific instructions about strategy. The task we set for them was to increase activity in the target area by as much and as consistently as possible.

We acquired fMRI data on a 3 Tesla Philips Achieva magnetic resonance imaging system (Philips Healthcare, Best, The Netherlands) using a single shot echo-planar imaging sequence (TR = 2 s, TE = 30 ms, 27 slices, 3 mm slice thickness, inplane resolution 2 mm×2 mm, soft tone mode). Patients were instructed to keep head movement to a minimum and fixate the middle of the picture/thermometer display during visual presentation, avoiding eye movements.

For the neurofeedback, a continuous signal from the target area (updated every TR and thus every 2 seconds) was displayed using the picture of a thermometer whose dial indicated the amplitude of the fMRI signal in the target area. Changes in the amplitude were indicated as the percent of signal change, calculated using the current signal intensity value and comparing it with the average value determined from the rest period immediately preceding each upregulation block. The scaling of the thermometer was in steps of 0.05%, with a maximum value of 0.5% (see Fig. 1). A change of background colour every 20 s indicated to participants whether their task was to regulate (green background) or rest (yellow background). The online GLM was computed with one predictor for the regulation state, convolved with a haemodynamic reference function. The top one-third (defined by the t value for the contrast between the regulation predictor and baseline) of the voxels from the target region was used to compute the feedback signal. For runs in which participants failed to upregulate the target area during the regulation periods (negative percent signal change), another target area was selected for the next run, using the cluster with the strongest activation for the regulation predictor. This adjustment in the target area was necessary in 15/32 (47.9%) of the sessions after the first NF run, and in 4 sessions after the second run. The reasons for this approach were two-fold. First, the adjustment of ROIs aided the shaping of mental strategies in the desired direction. Shaping is a common concept in the operant learning of a highly demanding task [11]. Secondly, our focus was not so much on the ability of participants to learn to regulate a specific brain region but on the effects of the NF training procedure on participants’ mood.

### Control Procedure (IM group)

The control intervention used the same basic stimulation procedure as the NF intervention, but it was performed outside the scanner. Patients were instructed to engage in positive imagery strategies similar to those reported by the NF group and to evoke positive memories during the blocks on which the background screen was green, and to rest during the blocks on which it was yellow.

### FMRI Data Analysis

For offline analysis, we performed the customary steps in three-dimensional fMRI analysis using the BrainVoyager QX (Braininnovation, Maastricht, the Netherlands) software package. The data were preprocessed using motion correction, temporal high pass filtering (2 sine/cosine pairs, or 0.005 Hz) and smoothing (3 s) and spatial smoothing (6 mm), following procedures described elsewhere [9]. For the region-of-interest (ROI) analysis, beta values and *t*-statistics for the upregulation predictor were extracted for the neurofeedback runs for each target ROI in order to obtain a measure of the participant’s self-regulation performance. The *t*-values were then entered into a 2-way repeated measures ANOVA (run: 3 levels; session: 4 levels) with subject as random factor. We also computed a 2-way ANOVA with the same factors on the whole-brain data in Talairach space in order to identify the overall network supporting the NF training task. Thresholds were identified for the whole brain maps at *p*<.05 voxelwise, with cluster correction at *p* = .05, using the cluster-level correction algorithm implemented in Brainvoyager to correct for multiple comparisons [24]. (For individual contrasts in the ANOVA, a more stringent threshold of *p*<.001 was used, corrected for multiple comparisons in the same way as the whole brain maps.).

### Psychometric Data Analysis

The psychometric data were analyzed in SPSS 16.0 (SPSS Inc., Chicago, IL, USA) with *t*-tests or analysis of covariance (ANCOVA) as appropriate. All the variates tested as being approximately normal (Kolmogorov-Smirnoff test, all *p*s >.3).

### Correlation Analysis

Correlations between each patient’s up-regulation improvement, as defined by a subtraction of the average *t*-value during the final session from the first session, and improvement on the HDRS, POMS and PANAS subscales were computed using Spearman’s rank correlation test.

## Results

We allocated 16 patients with a diagnosis of recurrent depression to a neurofeedback (NF, *N* = 8) or an imagery control (IM, *N* = 8) group. Patient groups were matched for demographic characteristics and clinical parameters (Table 1). The groups were also identical in terms of the severity of depressive symptoms before the intervention (HDRS-21 mean: NF = 18.125; IM = 17.75, *t*(14) = 0.15, *p* = .89; HDRS-17 mean: NF = 14.375; IM = 13.88, *t*(14) = 0.23, *p* = .82) and on reward sensitivity (BIS/BAS) and metacognitive measures (TCQ, TCAQ) (all *p*s >.1).

### Neurofeedback Success

**Figure 2:**
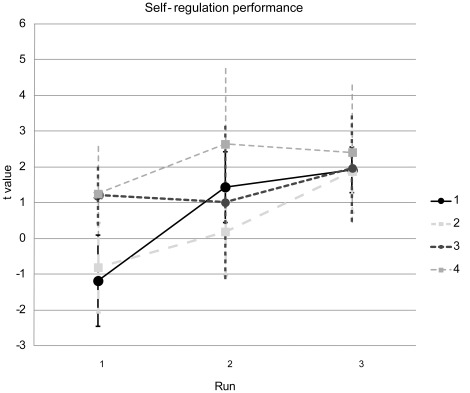
Neurofeedback success. Although self-regulation performance was varied during the first run (indicated by the low *t* values, scaled on the y-axis), participants achieved reliable upregulation during runs 2 and 3, with more stability in the later sessions. Data points represent group means and error bars represent the SD.

Patients in the NF group successfully learned to upregulate the target area, as indicated by a significant intercept in a repeated-measures ANOVA (*F* [1], [7] = 6.88, *p* = 034) (Fig. 2). The effect of run was significant (*F* [2], [14] = 4.08, *p* = 04), but neither the effect of Session nor the Session x Run interaction was significant (*p*s>3). The effect of run was produced by a linear increase from Run 1 to Run 3 (linear contrast: *F* [1], [7] = 5.72, *p* = 048). The Session x Run interaction for the linear contrast was marginally significant (*F* [1], [7] = 4.31, *p* = 076), reflecting the steeper increase in the first two sessions compared to Sessions 3 and 4. The target areas (which could comprise more than one anatomical region) were in the right (28 runs) or left (34 runs) ventrolateral prefrontal cortex (VLPFC), the left (29 runs) or right (19 runs) insula, the left (11 runs) or right (11 runs) dorsolateral prefrontal cortex (DLPFC), the left (2 runs) or right (1 run) medial temporal lobe or the orbitofrontal cortex (1 run), regions strongly implicated in the control of emotions [25]. Since it was required to adjust the target ROI during a substantial number of runs two additional analyses were performed. Firstly, it was investigated how up-regulation affected the initial ROIs that were selected during run 1. The effects of both run and session were not significant (*p*>3 and *p*>2 respectively) and the Session x Run interaction showed a trend towards significance (*F* [6], [42] = 1.89, *p* = 106). Secondly, we investigated whether a learning curve was present for the first neurofeedback run of each session and found a significant positive linear trend (*F* [1], [7] = 7.077, *p*<05), indicating improved control across sessions of the ROI that was selected based on the functional localizer.

### Clinical and Psychometric Measures

**Figure 3:**
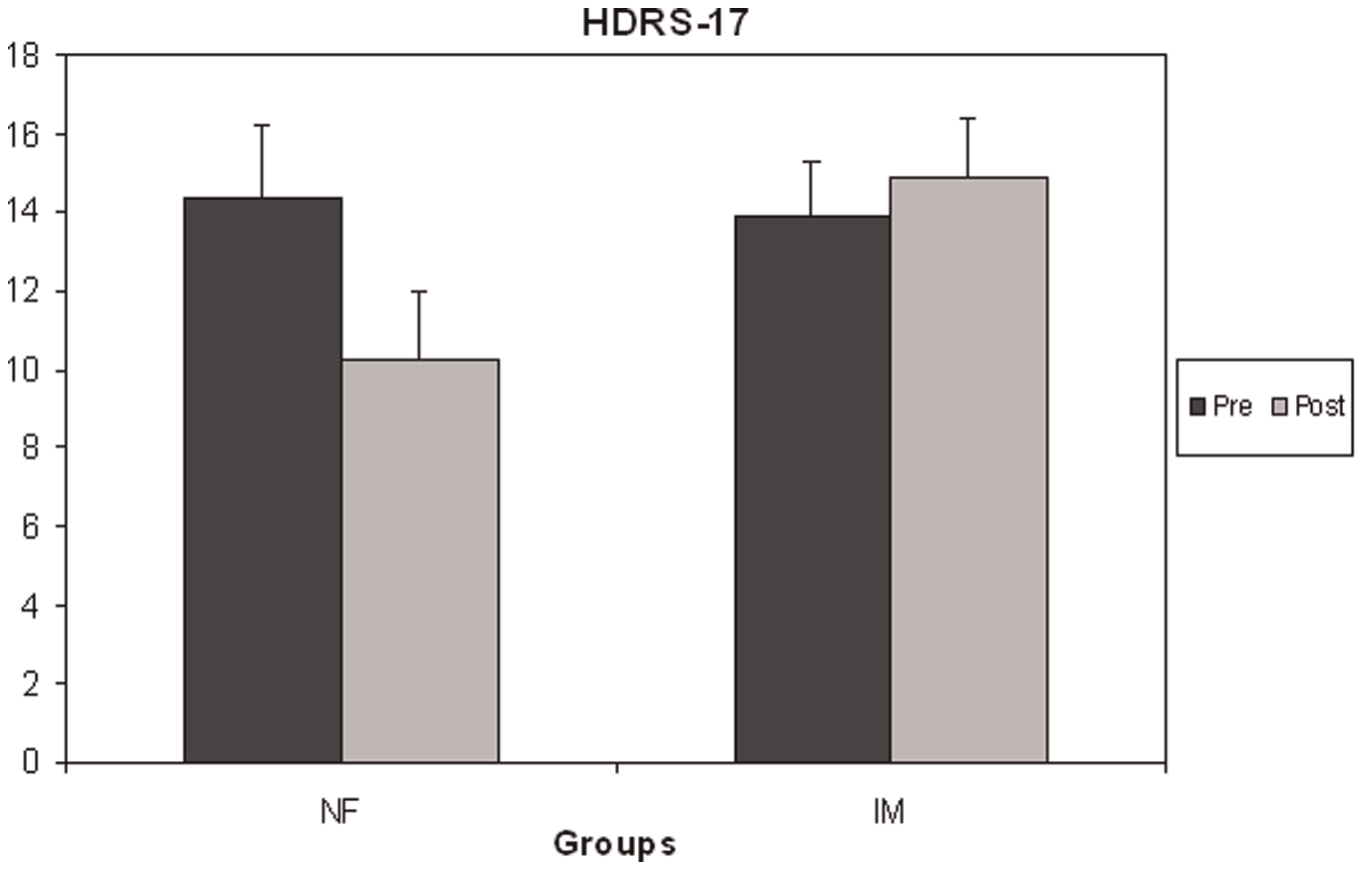
Neurofeedback produced clinical improvement that was not seen in the control group. Patients in the neurofeedback (NF) treatment group, but not those in the imagery (IM) control group, improved significantly on the 17-item Hamilton Depression Rating scale, a standard clinical measure of depression severity and treatment effects. Lower values denote clinical improvement (error bars: standard errors).

The NF group showed significant clinical improvement on the HDRS-17 (Fig. 3). A repeated-measures ANOVA with the factors Time (pre/post-intervention) and Group (NF/IM) yielded a significant interaction (*F* [1], [14] = 10.15, *p* = 007). To rule out an effect of gender imbalance across groups the same analysis was repeated with the factor Gender included as covariate, and similar results were obtained (*F* [1], [13] = 9.36, *p* = 009). The HDRS-17 scores of patients in the NF group decreased significantly (4.13 points (SD = 2.75) from a mean of 14.38 to 10.25, *t*(7) = 4.24, p = .004), but the change in the IM group (from 13.88 to 14.88) was not significant, *t*(7) = −0.78, *p* = 46). The effect size (Cohen’s *d*) of the improvement from treatment in the NF group was 1.5. Before the intervention, all patients had scores >8, but after the intervention, two of the NF patients had remitted (HDRS-17<8 [26]), and three additional NF patients (and one patient in the IM group) had scores of 8, thus fulfilling the criterion used in CBT trials for full treatment response [27]. Whereas clinical improvement was confined to the NF group, both groups showed within-session improvement in current mood on the POMS. This effect was supported by a significant intercept in a 2-way repeated-measures ANOVA performed on the difference scores (*F* [1], [14] = 21.7, *p*<001). After correcting for Gender and TMD baseline (TMD pre-test session 4– session 1) no significant effects (*p*s>1) were found apart from a significant Session x TMD baseline interaction (*F* [3], [36] = 6.60, *p* = 001), indicating that the size of TMD improvement decreased over sessions and with reduced TMD baseline scores. On PANAS NA difference scores, the NF group was significantly lower than the IM group (*F* [1], [14] = 16.18, *p*<001), indicating that the NF group decreased their NA scores more than the IM group. However, neither the Session effect nor the Group x Session interaction was significant (*p*s>7). The inclusion of Gender and PANAS NA baseline (PANAS NA pre-test session 4– session 1) as covariates did not alter any of these results (Group effect (*F* [1], [12] = 17.95, *p = *001), Session effect (*p*s>4), Group x Session interaction (*p*s>5)). A significant interaction was found between Session and PANAS NA baseline (*F* [3], [36] = 13.66, *p*<001). An ANOVA yielded no significant effects for the PA scores (*p*s>2), and adding Gender and PANAS PA baseline (PANAS PA pre-test session 4– session 1) as covariates only returned a significant Session x PANAS PA baseline interaction (*F* [3], [36] = 40.29, *p*<001).

The significant Session x Baseline score interactions were further investigated. For this purpose, patients were divided into a high improvement group (scores > median) and low improvement group (scores < median) and the effect of Session was tested. A significant Session x Improvement group interaction was found for TMD (*F* [3], [42] = 8.20, *p*<001), PANAS NA (*F* [3], [33] = 5.81, *p* = 003) and PANAS PA (*F* [3], [33] = 7.69, *p*<001). These interactions were driven by the finding that the improvement groups that showed the most within-session improvement during early sessions showed a significantly linear decreased within-session improvement over session. For PANAS PA and NA this trend was shown by the low improvement group ((*F* [1], [6] = 10.49, *p* = 018) and (*F* [1], [6] = 6.81, *p* = 04) respectively), for TMD by the high improvement group (*F* [1], [7] = 22.22, *p* = 002).

A significant positive correlation was found between up-regulation improvement and improvement on HDRS (*r* = 747, p = 033). Thus, the better a patient was at up-regulating the target area during the final session in comparison to the first, the more points a patient improved on the HDRS. No significant correlation was found between up-regulation improvement and POMS (*p*>7) or PANAS PA (*p*>7) or NA (*p*>4).

### Whole-brain fMRI Results

**Figure 4:**
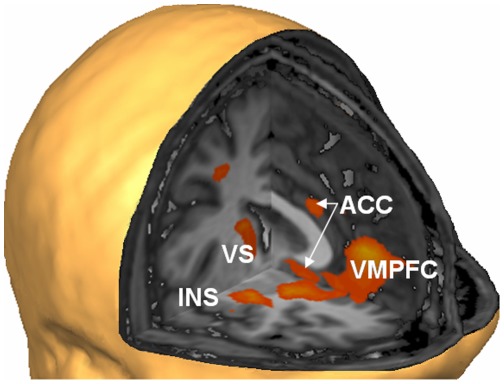
The localiser procedure identified networks of positive mood. Higher activation of right insula (INS), ventral striatum (VS), anterior cingulate cortex (ACC) and ventromedial prefrontal cortex (VMPFC) during presentation of positive compared to neutral images in the localiser runs (for full list of areas see Table 2). The localiser runs were effective in identifying brain areas responsive to positive images, which were used as target regions of interests (ROIs) for the subsequent neurofeedback procedure. The figure shows the contrast map thresholded at p<.05 (cluster level corrected) on a sample brain seen from the right and front (Talairach coordinates of virtual cuts: x = 0, y = 0, z = −2).

Group analysis of the contrast between conditions with positive and neutral images (Table 2, Fig. 4) in the localizer scans yielded activation in the bilateral VLPFC/insula region, which is consistent with its prominence in the individual contrast maps producing the target areas. Additional areas with higher activation to positive images included the ventromedial PFC, parts of the cingulate cortex, regions in the bilateral DLFPC and bilateral parietal cortex, and higher visual areas.

**Table 2:** Areas activated for contrast “positive” vs. “neutral” images in the group map of the localizer procedure.

Anatomical label	Talairach coordinates (x/y/z)	Cluster size (mm^3^)
R IPL	50/−26/40	3129
R IT	43/−65/−6	6215
R POJ	30/−76/26	4962
Bil VMPFC/VLPFC (including Bil insula)	−2/36/4	26032
R DMPC	30/−3/44	1048
R DLPFC	19/40/44	1500
R Caudate nucleus	14/0/15	691
ACC	3/23/26	1032
L DLPFC	−16/40/40	2629
L POJ	−22/−80/26	1114
L DLPFC	−33/9/46	2628
L Lentiform nucleus	−19/−6/12	852
L IPL	−43/−38/32	6724
L EVC	−43/−68/0	6792
L DLPFC	−39/40/20	922

Abbreviations: R  =  right, L  =  left, Bil  =  bilateral, ACC  =  anterior cingulate gyrus; D/VLPFC  =  dorso/ventrolateral prefrontal cortex; D/VPMC  =  dorsal/ventral premotor cortex, EVC  =  extrastriate visual cortex; IPL  =  inferior parietal lobule, POJ  =  parieto-occipital junction; IT  =  inferior temporal cortex.

**Figure 5:**
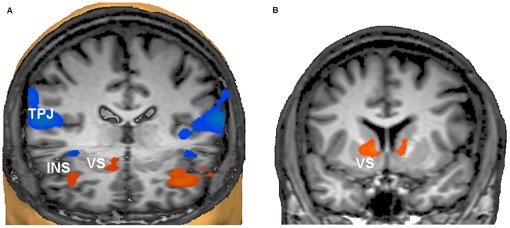
Network activation and deactivation during neurofeedback. a) Activation of the insular cortex (INS) bilaterally and the right ventral striatum (VS) supported the neurofeedback task, whereas the temporoparietal junctions (TPJ) of both hemispheres were deactivated. The TPJ is recognised as part of the brain’s “default mode network” that is deactivated during effortful tasks. For a full documentation of the activated and deactivated networks see Table 3. View from the front and above. The right side of the brain is on the observer’s left (Talairach coordinates of virtual cuts: y = 25, z = −2). b) Successive training sessions produced further increases of activation during upregulation periods in the VS bilaterally (coronal view at y = 7, the right side of the brain is on the observer’s left).

Activation increases during upregulation periods of the neurofeedback scans included but were not confined to the individual target regions. Rather, the group map for the upregulation predictor showed activation of the bilateral anterior insula and hippocampal regions, bilateral medial premotor and prefrontal regions, the right ventral striatum, and the left cuneus (Fig. 5a, Table 3). Deactivation was prominent in the bilateral temporoparietal junction (TPJ), and it extended into the posterior insula, early and higher visual areas, and the right DLPFC (Fig. 5a, Table 3). Significantly higher activation in the upregulation periods of the late (Weeks 3 and 4) compared to early (Weeks 1 and 2) sessions was observed in the bilateral ventral striatum, and in left extrastriate visual cortex (*p*<001, cluster level corrected; see Fig. 5b and Table 3).

**Table 3:** Neurofeedback-related activation and deactivation (group whole-brain analysis).

a) Areas activated during upregulation periods				
Anatomical label		Talairach coordinates (x/y/z)		Cluster size (mm^3^)
L IFG/insula		−39/17/14		20960
L cuneus		−17/−52/9		10825
L HC		−28/−29/−7		6548
R PHG		30/−47/2		3397
R insula		25/19/16		4077
R VS		9/6/0		1294
L DMPFC		−13/43/36		3708
Bil Medial frontal gyrus		−5/7/48		1643
**b) Areas deactivated during upregulation periods**				
**Anatomical label**		**Talairach coordinates (x/y/z)**		**Cluster size (mm^3^)**
R TPJ		52/−48/24		24077
L TPJ		−54/−27/20		15250
R DLPFC		39/27/33		12355
R posterior insula		33/−13/6		1918
R EVC		32/−89/1		3787
Bil PVC		−9/−72/10		35879
**c) Areas with increased activity during late compared to early session**				
**Anatomical label**	**Talairach coordinates (x/y/z)**		**Cluster size (mm^3^)**	
Bil VS	−11, 9, 5 13, 15, 6		1073 969	
L EVC	−33, −79. −7		6298	

Abbreviations: See Table 2; additionally: DMPFC  =  dorsomedial prefrontal cortex; HC  =  hippocampal complex; IFG  =  inferior frontal gyrus; PHG  =  parahippocampal gyrus; PVC  =  primary visual cortex; TPJ  =  temporo-parietal junction; VS  =  ventral striatum; EVC  =  extrastriate visual cortex.

### Neurofeedback Strategies Debriefing

Patients in the NF group reported initially using imagery of the positive scenes in the localizer scan in an attempt to increase activation in the target brain areas, but they later changed to evoking memories and imagery of autobiographically relevant material. For example, the happy memories that they reported as successful strategies included holidays, thoughts about their family being happy, and imagery of beautiful scenes from nature. Some patients attained good self-regulation of the target areas through mental simulation of future successes, and one patient successfully used imagery of an out-of-body experience. Conversely, during rest periods, the patients reported trying to “empty their thoughts” and to meditate. Patients in the IM group were instructed to engage in similar strategies as those reported by the NF patients. At debriefing, they confirmed that they had used these strategies. No patient reported any distress arising from the procedure.

## Discussion

In the present study, four sessions of non-invasive fMRI-neurofeedback reduced the symptoms of depression with an effect size similar to those obtained with deep brain stimulation (DBS) [3]. Although the mental strategies of positive thoughts, memories, and imagery may have played a considerable part in this improvement, the neurofeedback procedure was crucial as evidenced by the absence of any clinical improvement in the control group. This effect of the neurofeedback intervention can be a result of several factors, including the self-regulation of emotion networks, but also non-specific effects of reward experience and scanner environment. These potential confounds and strategies for overcoming them in future studies will be discussed below. The lack of improvement in the control group may seem surprising at first, considering the often reported placebo responses in drug trials. However, enrolment in drug trials raises very different expectations to a brief emotion regulation intervention, and control or waiting list groups of psychotherapy trials often show no improvement at all [28]. In addition to the feasibility of the technique in patients with depression, which is important in light of the often reported motivational deficits in this patient group, this study thus shows encouraging clinical effects, which need to be corroborated in clinical trials.

The significant interactions between Session on the one hand and baseline TMD and PANAS on the other hand showed that within-session improvements on the POMS and PANAS are influenced by the improvement over time, which can result in ceiling effects. However, none of these factors interacted with Group and thus different sensitivity to the mood measures does not seem to have influenced the key results of our study. Whereas groups did not differ in the immediate mood effect as measured with the POMS, a questionnaire that does not capture the full syndrome of depression, only the NF group improved significantly on the HDRS, which captures more sustained clinical effects, after treatment. This suggests that the neurofeedback procedure mainly enhanced the consolidation of the patients’ improved control over their mood states. This interpretation would conform to a recent report that emotion regulation without neurofeedback resulted in immediate but not sustained effects at the neural level in patients with major depression [29]. The finding that patients who became better at up-regulating their target area also improved more on the HDRS supports this finding. The non-significant correlation between up-regulation improvement and POMS or PANAS was as expected as improvement on these scales leveled off after large improvements during early sessions and was also seen in the control group. One attractive interpretation of our results in the context of current cognitive models of depression is that the NF patients managed to activate positive cognitive schemas that were otherwise dormant [5]. The interplay of cognitive and operant strategies is also reflected in the pattern of areas that were activated or deactivated regardless of the specific target area (Fig. 5, Table 3). The activation of cognitive control areas in the prefrontal cortex and of the hippocampal complex would be compatible with the active selection of the appropriate autobiographical strategy for positive mood induction and cognitive appraisal of emotions, whereas activation of the ventral striatum, which increased during the later sessions, has been associated with both operant learning and rewarding experiences [30], [31]. The deactivation of the TPJ (equivalent to higher activation during the rest epochs) is compatible with the role of this area in the “default mode network” and its deactivation during attention-demanding processes [32]. The focal pattern of activation and deactivations during the upregulation blocks also makes it unlikely that the increasing activation of the target area was achieved by some non-specific physiological artifact. Nevertheless, to completely rule out this possibility, online monitoring of peripheral physiological parameters should have been conducted and future studies should incorporate these measures. The feasibility of fMRI-neurofeedback under online control for potential movement [41] or physiological changes [45] has been demonstrated. Because the control group was not scanned we do not know whether their execution of the imagery procedure involved similar patterns of brain activation. However, in a study with a similar design (albeit only one session) in healthy individuals where both groups were scanned we found clear differences in the training effects on the activation of the emotion network [9]. It is also worth noting that the initial target area identified for positive affective stimuli varied across sessions for the same participant, although it was mostly located in ventrolateral prefrontal cortex or the insula. Without a scanned control group we cannot say whether this reflects a changing emotion regulation during the neurofeedback procedure [33] or whether it merely indicates normal variability in the brain’s response to emotional stimuli over time [34]. We do not believe that the activation patterns found for emotion regulation in the present study primarily reflect patients’ medication because the few studies investigating the neural correlates of emotion regulation in unmedicated depressed patients obtained comparable results. Medication-free patients have shown increased activity in the insula and frontal regions and decreased activity in temporal and parietal regions during an emotion reappraisal task [35], [36] and decreased DLPFC activity on an emotional information processing task [37].

Conceptually, fMRI-based neurofeedback combines the principles of cognitive-behavioral therapy (CBT) with those of physical brain stimulation. Compared to electromagnetic brain stimulation techniques, it has the advantage of enhancing the patient’s self-efficacy [38], which is an important principle in cognitive restructuring. Neurofeedback combines biological and cognitive treatment principles in a way that differentiates it both from traditional biofeedback [39] and cognitive therapy and may therefore be particularly useful for patients who have not responded to or are reluctant to engage in psychological therapies. The feedback element and success control could constitute an incentive for patients who are not sufficiently motivated for standard psychotherapies. Another attraction of the fMRI-neurofeedback technique lies in its adaptability to individual target areas that may differ across individuals and time. However, in order to stabilize long-term benefits, homework assignments or booster sessions might be usefully added to the present protocol.

The present study was a proof of concept of the feasibility of neurofeedback and its potential clinical benefits in depression. One limitation of the present study was the absence of blinding and randomization, and randomized controlled trials are needed to corroborate the clinical benefits. Lack of randomization and sequential group allocation can induce demographic or procedural biases, which can only be partly controlled for by including them as confounds in a covariance analysis. Yet patients assigned to the NF or IM group were recruited via the exact same resources and showed no meaningful differences in demographics apart from the uneven gender distribution, which needs to be addressed in future studies in order to assess whether the effect generalizes to female patients. Moreover, the testing of both groups overlapped in time period and both groups engaged in a task that required the same mental strategies and time commitment.

It might be useful to use Quality of Life scales and self-reported clinical scales to capture the patients’ response to treatment without the potential biases of unblinded assessments. However, by their very nature, procedures with a strong cognitive component are impossible to execute in a completely blinded design. A potential effect of task setting, if any, would hamper the performance of patients in the NF group who carried out the task in a noisy and highly confined space. The clinical and functional improvement suggests that the effects of neurofeedback can overcome these suboptimal circumstances, thereby strengthening the plausibility of neurofeedback for alleviating depression. It could be argued that the exposure to a technologically advanced method may have boosted confidence in the neurofeedback method or otherwise created a placebo effect, thereby decreasing the HDRS scores of the experimental group only, although there is no reported evidence for clinical improvements from scanning alone [40]. Yet future studies need to keep task setting more similar between groups, especially with regard to the use of highly technical equipment. A “sham” feedback procedure that presents on average similar success signals as in the active group but is not contingent on actual brain regulation is a possibility, but it cannot be excluded that patients will notice the non-contingency of the feedback. In our recent fMRI-neurofeedback study in patients with Parkinson’s disease we did rule out non-specific effects of the scanner environment on functional improvement [41]. All previous studies incorporating a control group did not find an effect of sham feedback either [8], [13], [14], [42]–[45]. It is certainly possible, though, that the experience of successful self-regulation of brain activity, quite independent of the target area, produces beneficial clinical effects. This possibility needs to be addressed in future studies employing different target systems in the brain. Another neurofeedback variable that needs to be carefully considered in future trials is block duration. The significant difference between positive emotion up-regulation and rest suggests that a block length of 20 s was appropriate for our study, yet future studies might benefit from employing longer block durations in order to give patients more time to disengage from the mental processes utilized for upregulation.

Our study differed from most other neurofeedback studies in that we flexibly adjusted the target area for subsequent runs based on areas that were upregulated in the previous run. This procedure was intended to support the shaping of learned responses because of the limitation of scanning time and the need to minimize patient frustration. However, this procedure may also have made it more difficult for patients to find the optimal up-regulation strategy and may overestimate the within session learning effect. Another limitation is that we cannot isolate the effective mechanism or mechanisms of the neurofeedback procedure from the present study. Although our data suggest that neurofeedback of emotion networks is more effective than emotion regulation without brain signals, we cannot determine the relative contribution of the general experience of gaining control about brain activation and the specific areas modulated. This issue could be addressed in a further study using another target system in the brain in a control group.

FMRI-based neurofeedback is a holistic approach that overcomes bio-psychological dualisms. It is therefore perfectly compatible with current models of depression and other complex mental disorders. It can be used to help patients and researchers understand the neural and cognitive processes that underlie depression. Most importantly, however, if the clinical benefits are replicated in clinical trials it may prove to be the first therapeutic application of functional imaging in the field of mental health. We can expect that knowledge about the contribution of dysfunctional brain circuits to psychiatric symptoms will continue to accumulate, which opens up the possibility of developing therapeutic imaging protocols similar to the present one for a wide range of neuropsychiatric disorders.

In this proof of concept study we demonstrate that patients with depression can learn self-control of emotion-related brain areas through fMRI-based neurofeedback. This procedure had clinical benefits compared to a control group, which engaged in emotional imagery outside the scanner, but further formal testing in randomized trials with blinded assessments is needed in order to assess the clinical efficacy.
